# Adaptive Interference Cancellation of ECG Signals

**DOI:** 10.3390/s17050942

**Published:** 2017-04-25

**Authors:** Aifeng Ren, Zhenxing Du, Juan Li, Fangming Hu, Xiaodong Yang, Haider Abbas

**Affiliations:** 1 School of Electronic Engineering, Xidian University, Xi’an 710071, China; afren@mail.xidian.edu.cn (A.R.); ZhenxingDu@163.com (Z.D.); lijuan12388@163.com (J.L.); fangming95@163.com (F.H.); 2 King Saud University, Riyadh 11653, Saudi Arabia; hsiddiqui@ksu.edu.sa; 3 National University of Sciences and Technology (NUST), Islamabad 44000, Pakistan; 4 Department of Computer Sciences, Florida Institute of Technology (FIT), Melbourne, FL 32901, USA

**Keywords:** ECG signal, interference cancellation, LMS algorithm

## Abstract

As an important biological signal, electrocardiogram (ECG) signals provide a valuable basis for the clinical diagnosis and treatment of several diseases. However, its reference significance is based on the effective acquisition and correct recognition of ECG signals. In fact, this mV-level weak signal can be easily affected by various interferences caused by the power of magnetic field, patient respiratory motion or contraction, and so on from the sampling terminal to the receiving and display end. The overlapping interference affects the quality of ECG waveform, leading to the false detection and recognition of wave groups, and thus causing misdiagnosis or faulty treatment. Therefore, the elimination of the interference of the ECG signal and the subsequent wave group identification technology has been a hot research topic, and their study has important significance. Based on the above, this paper introduces two improved adaptive algorithms based on the classical least mean square (LMS) algorithm by introducing symbolic functions and block-processing concepts.

## 1. Introduction

As the most important organ in the human body, the heart is the power source of metabolism of various organs and tissues. The basic units of the heart are the cardiomyocytes, and the human electrocardiogram (ECG) signal is actually their electrical activity response [1]. When there is a certain way to stimulate a certain intensity through the myocardial cells, it will result in intracellular and extracellular ion flows, resulting in an action potential. ECG signals completely record this change process [2], and ECG waveform reflects the physiological conditions of various parts of the heart in medical diagnosis, and is a very valuable reference for treatment [3].

The amplitude of the ECG signal is in the range of 10 μV to 4 mV and it is very small and weak and therefore very sensitive to the effects of various disturbances [4]. One of the main sources of interference are loop factors and 50 Hz frequency interference caused by the electromagnetic field and the patient, baseline wander (BW) caused by the patient’s limb movement and breathing, and electromyogram (EMG) interference caused by skeletal muscle stimulation and contraction [5].

**Figure 1:**
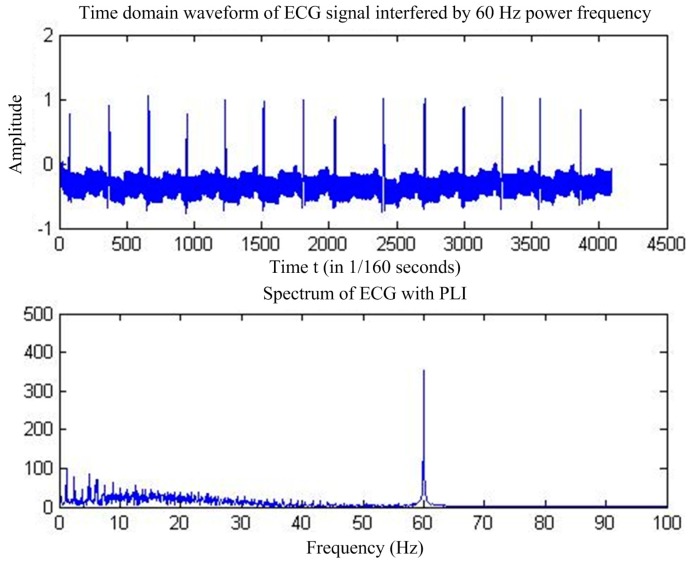
ECG signal with PLI.

Power frequency interference [6], the most common ECG signal interference, is caused by the magnetic field distributed by the human body by the power supply, resulting in added 50 Hz sinusoidal and harmonic components in the pure ECG signals [7]. Figure 1 shows the time-domain and frequency-domain diagrams of the ECG signals with power-line interference (PLI).

**Figure 2:**
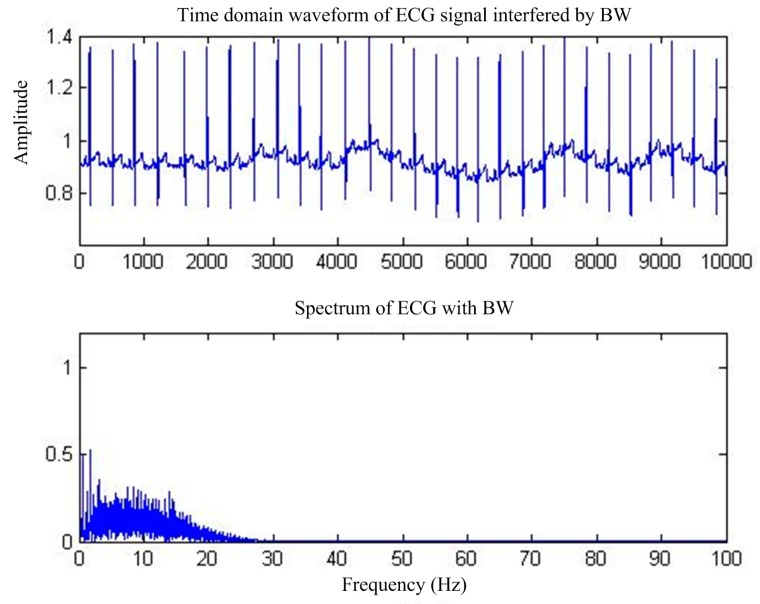
ECG signals under BW interference.

BW is another interference affected by the patient’s ECG monitored breathing, electrode movement, and other frequently occurring factors [8]; its shape is similar to a periodic sinusoidal signal, generally below the 1 Hz low-frequency signal, resulting in a baseline signal instability drift, making the ECG waveform show a slow change [9]. The time and frequency domains of the ECG waveforms affected by BW are shown in Figure 2.

**Figure 3:**
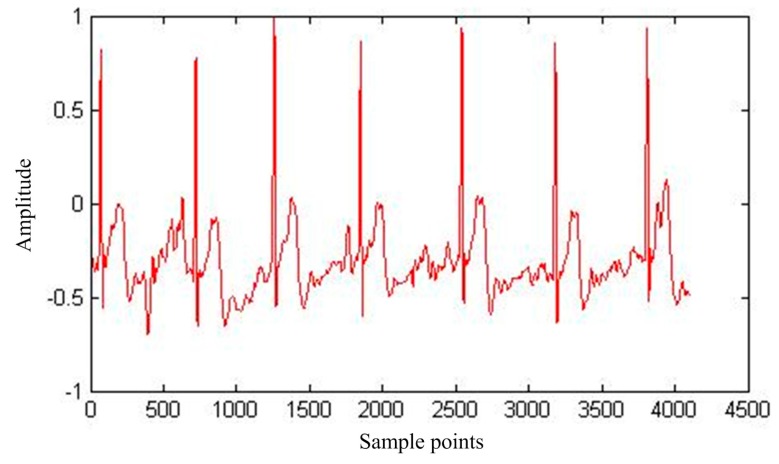
ECG signals with no interference.

When the ECG signal is collected using different measurement modes, the signal strength is different. In the actual acquisition in front of the armpit the ECG signal is the most obvious, as the ECG signal is obtained with no interference, as shown in Figure 3.

Based on the classical least mean square (LMS) algorithm [10], two improved adaptive algorithms are proposed, namely, the normalized LMS (NLMS) algorithm [11] based on symbol function and the normalized block-processing LMS (BLMS) algorithm based on symbol function [12]. The symbolic functions [13] and the block-processing [14] concept are introduced and applied to the the elimination of two kinds of interference: ECG signal frequency interference and BW interference. The MIT-BIH ECG database and the real ECG data collected by a miniature ECG collector in the laboratory were used to validate the algorithm and analyze the results in detail.

## 2. Proposed Technique for Adaptive Interference Cancellation

### 2.1. Adaptive Noise Cancellation Based on LMS Algorithm

**Figure 4:**
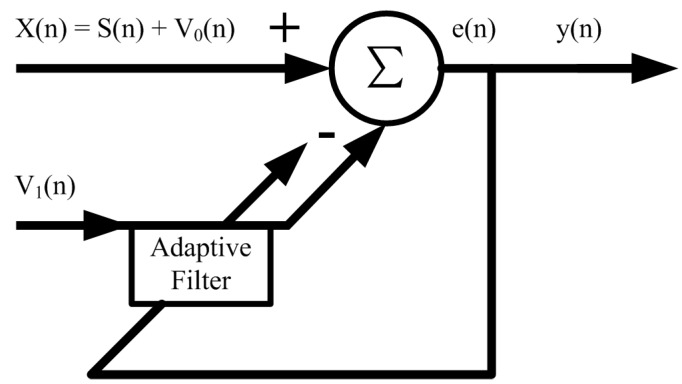
Block diagram of adaptive noise cancellation.

The basic principle of the adaptive noise canceller is to use the noise source to output, then to digitally filter [15], to estimate the noise most accurately, and then to subtract the estimated noise from the original input, thus achieving the separation of the pure useful signal from the noise. Figure 4 shows a block diagram of an adaptive noise canceller based on Wiener filtering [16], wherein the main input signal is X(n), which consists of the useful signal s(n) and the background interference signal v_0_(n), and these two are not related. The reference input signal v_1_(n) must be the input signal associated with the interfering signal v_0_(n).

Because the reference signal v_1_(n) is correlated with the interference portion v_0_(n) in the main input, the filter will remove this correlation at its output. This is achieved by generating an estimate of the interference in the main input signal from the noise of the reference channel and subtracting the estimated interference value from the main input to obtain the final output of the system as an estimate of the wanted signal. In summary, the specific steps for implementing adaptive noise cancellation based on LMS can be summarized as follows:

1. The initial value is set to start the default weight coefficient vector.
2. Calculate the output signal of the adaptive FIR filter, wherein the order is *L* − 1:
  (1) $$\hat{v}(n)=\sum_{i=0}^{L-1}W_{i}v_{1}(n-i),$$
3. Estimate the error of the current time n:
  (2) $$e(n)=x(n)-\hat{v}(n)\approx \hat{s}(n),$$
4. Use the steepest descent LMS algorithm to adjust the weight vector of the filter continuously:
  (3) $$W_{i+1}(n+1)=W_{i}(n)+2\mu e(n)X(n-i)\;0\leq i\leq L-1,$$
5. Verify whether the standard deviation of the standard error has been satisfied. If it is, immediately stop iteration; otherwise, continue to the following operation.

### 2.2. NLMS Algorithm Based on Symbol Function

The NLMS algorithm is a kind of adaptive algorithm that is extended and improved based on LMS algorithm. The improvement of the algorithm is to use the variable step method, thus reducing the time required for full convergence, which corresponds to the weight coefficient update formula:

(4) $$W(n+1)=W(n)+\left[\frac{\mu }{p+X^{T}(n)X(n)}\right]e(n)X(n),$$

Therefore, the variable step size can be expressed as:

(5) $$\mu_{e}(n)=\frac{\mu }{p+X^{T}(n)X(n)},$$

Here, the step size is a fixed factor that controls the speed of convergence of the algorithm. The parameter p prevents the denominator from being too small. When the step size parameter is too large, the p value is generally a small positive number [17].

One of the major drawbacks of the adaptive algorithm extended by the basic LMS algorithm is that the excess mean square error is too large to cause distortion of the filtered signal. Thus, Muhammad proposed an error NLMS (ENLMS) algorithm with a variable step size proportional to the square of the error signal in the adaptive algorithm for removing the ECG signal power frequency interference. The corresponding iterative adjustment formula of the algorithm is as follows:

(6) $$W(n+1)=W(n)+\left[\frac{\mu }{p+e^{T}(n)e(n)}\right]e(n)X(n),$$

Therefore, in the LMS algorithm, the variable step size is expressed as:

(7) $$\mu_{e}(n)=\frac{\mu }{p+e^{T}(n)e(n)},$$

The parameters *μ* and *p* have the same meanings as above. Compared to the NLMS algorithm, ENLMS in the step-length selection is no longer dependent on the input signal; thus, for the convergence rate and steady-state error, ENLMS should perform better.

However, according to the formula of the variable step size, the ENLMS algorithm needs additional computation to obtain the variable step size compared to the traditional LMS algorithm. Therefore, to further reduce the amount of computation without affecting the quality of the filtered signal, a sign function is introduced in the weight coefficient updating Equation (9).

The symbolic function sgn(x) is defined as follows:

(8) $$\mathrm{sgn}(n)=\left\{\begin{array}{l} 1:x>0\\ 0:x=0\\ -1:x<0 \end{array}\right\},$$

An NLMS algorithm based on the symbolic function is obtained. Therefore, the final weight coefficient iterative adjustment formula is as follows:

(9) $$W(n+1)=W(n)+\mu_{e}(n)\mathrm{sgn}\left\{X(n)\right\}\left\{e(n)\right\}.$$

### 2.3. Normalized BLMS Algorithm Based on Symbol Function

From the NLMS algorithm based on symbol function proposed in the previous section, the concept of block processing can be introduced to further reduce the computational complexity of the algorithm.

The basic idea of the widely used BLMS algorithm [11] is that, unlike the basic LMS algorithm, the filter coefficients are recalculated and updated for each sample value. The block-processing algorithm is implemented in each block region, that is, every k points to a weight coefficient update [18]. Therefore, the filter weight vector iterative adjustment formula of the block-processing algorithm is as follows:

(10) $$W(n+1)=W(n)+\mu \sum_{i=0}^{k-1}X(nk+i)e(nk+i),$$

Then, the time-averaged gradient vector is:

(11) $$\phi (n)=\mu \sum_{i=0}^{k-1}X(nk+i)e(nk+i),$$

The filter coefficient vector based on the BLMS algorithm is adjusted once per k sampling points, and the coefficient update is based on the average gradient vector of the k sampling points. It can be deduced that the BLMS algorithm also converges to the Wiener solution, but this does not mean that it will achieve the final result sooner than the LMS.

The block processing is introduced into the NLMS algorithm based on symbol function proposed in the previous section, that is, the input signal is processed in blocks (10), and then the absolute value of the error signal in each block region of length L is selected. The value is used to obtain the variable step size. Thus, the weight coefficient updating formula becomes:

(12) $$W(n+1)=W(n)+\frac{\mu }{e_{Li}^{2}}\mathrm{sgn}\left\{X(n)\right\}\mathrm{sgn}\left\{e(n)\right\},$$

Then, we obtain the weight coefficient update formula of the normalized BLMS algorithm based on symbol function, which is further simplified, where $e_{L_{i}}=\max\left\{\left|e_{k}\right|,k\in Z_{i}^{\prime }\right\},Z_{i}^{\prime }=\left\{iL,iL+1,\ldots ,iL+L-1\right\},i\in Z$, and *if* $e_{L_{i}}=0$, Equation (12) becomes:

(13) W(n+1)=W(n).

## 3. Implementation

### 3.1. Adaptive Interference Cancellation to Remove Power Frequency Interference 

In the following, we use the MIT-BIH public library’s record file and the data collected by the minicollector to compare and validate the ECG signal’s interference elimination performance.

**Figure 5:**
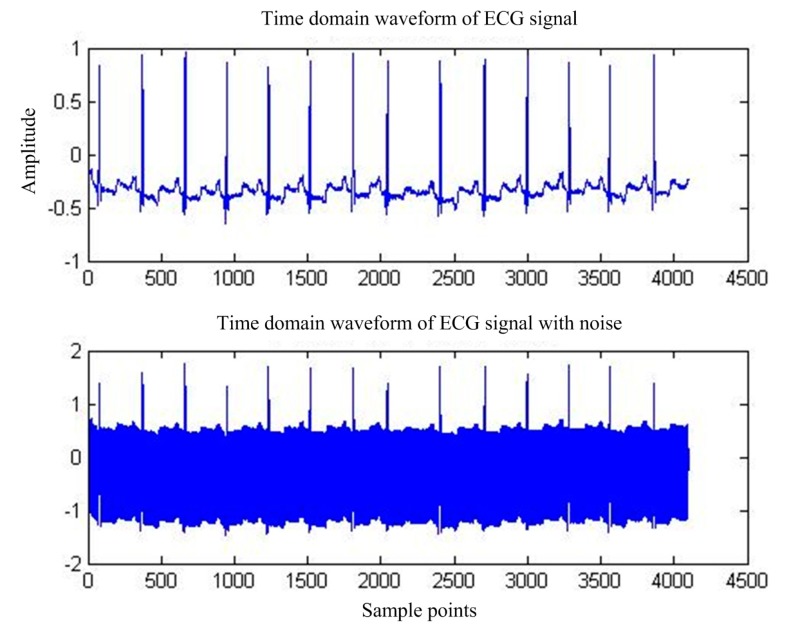
MIT-BIH ECG waveform before and after PLI.

**Figure 6:**
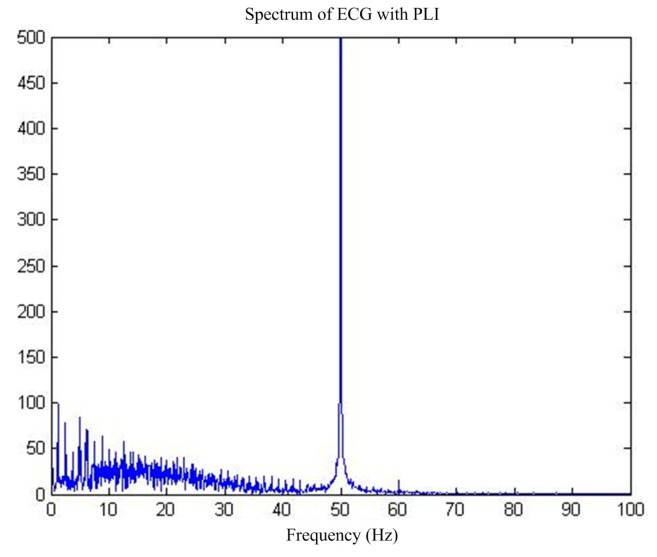
Spectrum of MIT-BIH ECG by PLI.

First of all, for the pure ECG signal from the MIT-BIH public library, artificially joining the same sampling rate of 50 Hz power frequency interference will add interference after the signal as the main filter input, the reference input, and select interference with the same frequency cosine [14]. Figure 5 shows the pure ECG signal and the waveform after superimposed PLI. Figure 6 shows the spectrum of ECG with PLI. It is clear that there is a strong 50 Hz noise.

**Figure 7:**
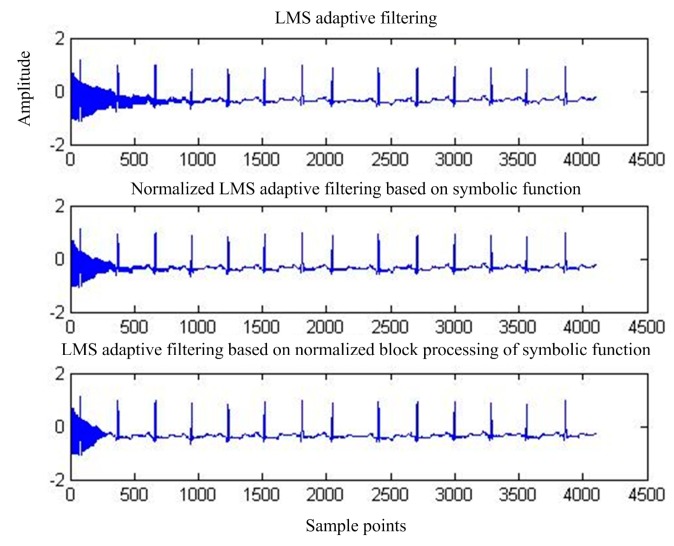
Output of the three adaptive interference cancellation algorithms.

**Figure 8:**
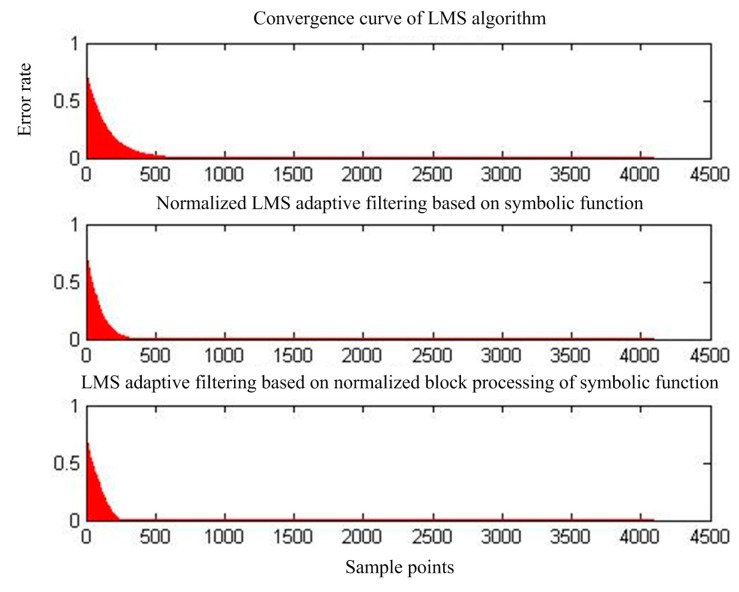
Mean square error convergence of the three adaptive interference cancellation algorithms.

Figure 7 shows the output of adaptive interference cancellation. Figure 8 shows the convergence of the statistical mean square error of the three adaptive interference cancellation algorithms.

**Figure 9:**
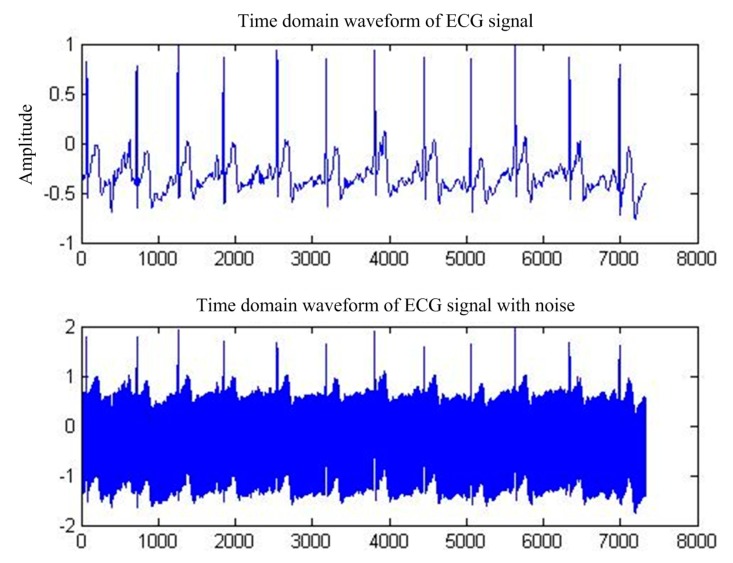
Before and after the impact of PLI by the ECG waveform.

**Figure 10:**
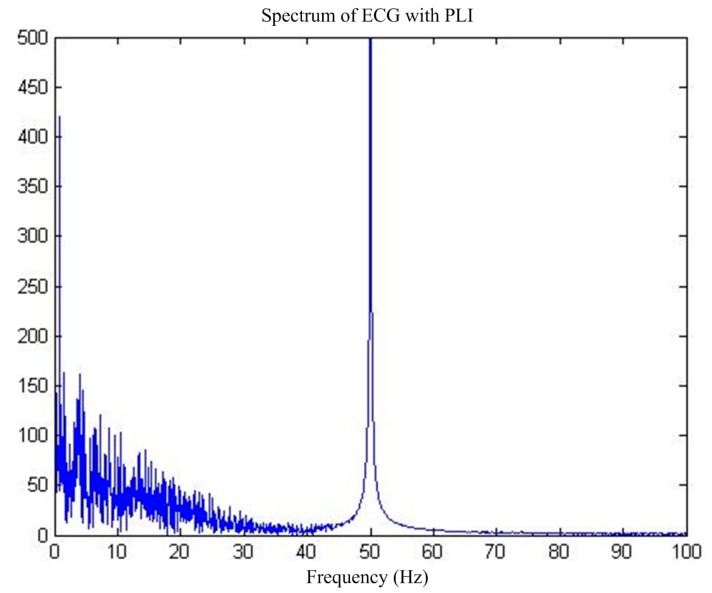
Influence of PLI on the ECG.

Based on the algorithms of ECG data acquisition in the MIT-BIH public library, the ECG data of a classmate were processed using a mini-ECG collector. Figure 9 shows the original ECG waveforms and the waveforms after adding PLI. Figure 10 is its spectrum.

**Figure 11:**
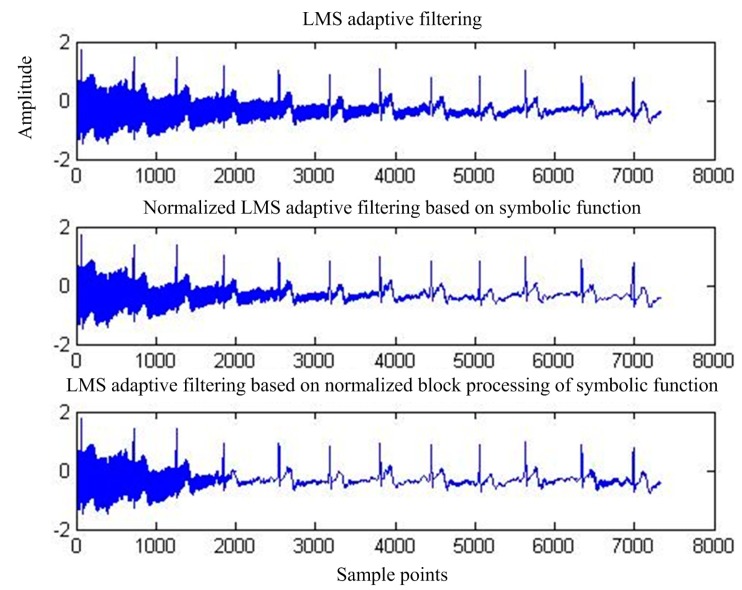
Output of the three adaptive interference cancellation algorithms.

**Figure 12:**
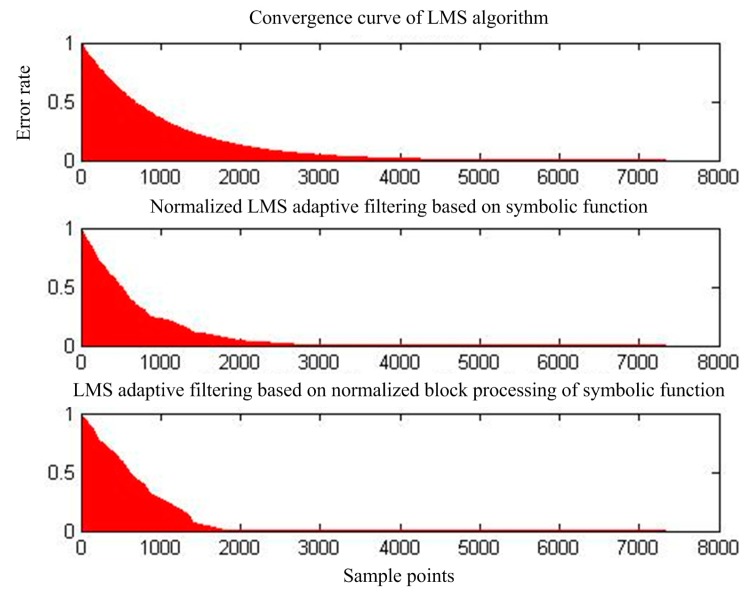
Convergence of mean square error of the three adaptive interference cancellation algorithms.

Figure 11 shows the output of adaptive interference cancellation. Figure 12 shows the average mean square error convergence curve for 100 simulation simulations of the three adaptive interference cancellation algorithms.

### 3.2. Adaptive Interference Cancellation Removes BW

**Figure 13:**
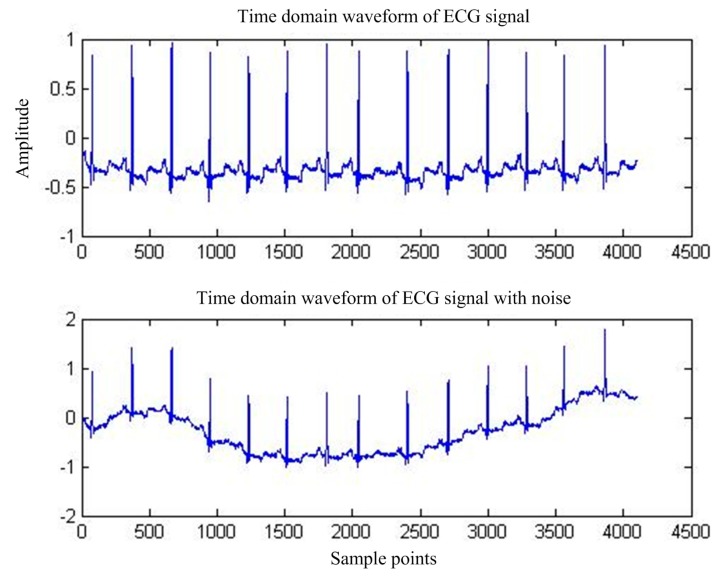
MIT-BIH ECG waveform before and after BW.

**Figure 14:**
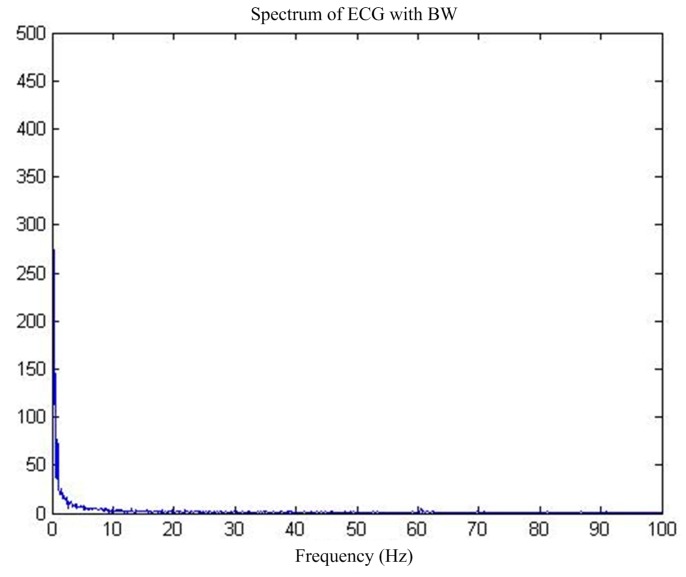
MIT-BIH ECG before and after BW.

First of all, for the pure ECG waveform sampled from the MIT-BIH common library, the baseline drift interference in the common library with the same sampling rate is superimposed on the pure ECG waveform, and then the mixed signal is used as the main input and the reference input is used as the artificial input of the baseline drift interference. Thus, the correlation between the reference input and the interfering signal in the main input is 1, and interference cancellation is most effective. Figure 13 shows the pure ECG waveform and the waveform after adding the baseline drift. Figure 14 is the spectrum of the waveform after adding the baseline drift. It can be seen that the baseline drift interference mostly concentrates on the low-frequency band near the zero frequency.

**Figure 15:**
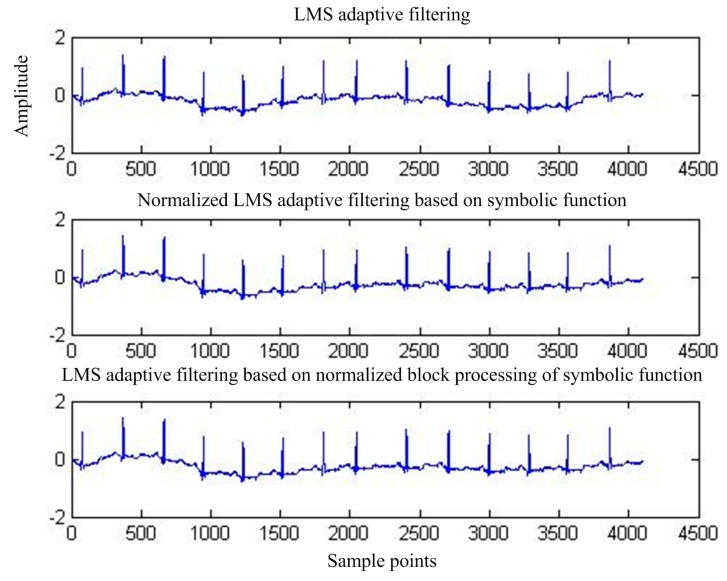
Output MIT-BIH signal after adaptive interference cancellation.

**Figure 16:**
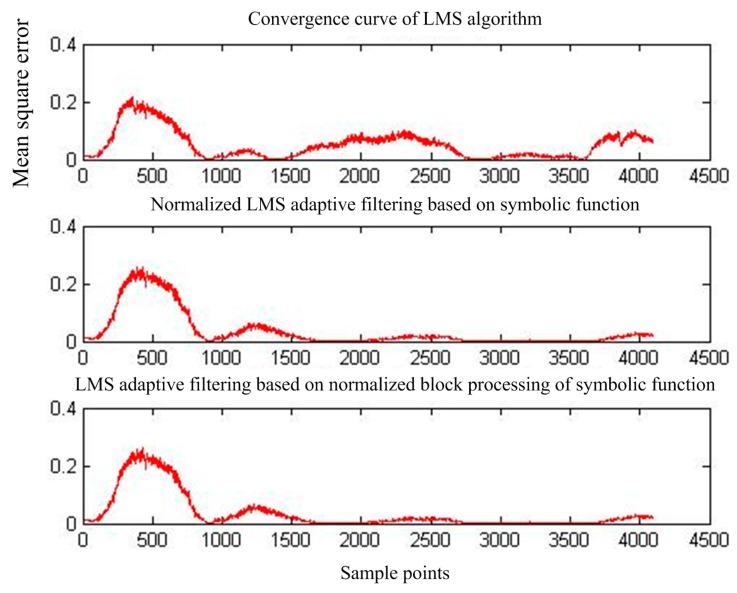
Mean square error convergence of the three adaptive interference cancellation algorithms for MIT-BIH signals.

Figure 15 shows the output of adaptive interference cancellation. Figure 16 shows the convergence curve of the statistical mean square error of the three adaptive interference cancellation algorithms.

**Figure 17:**
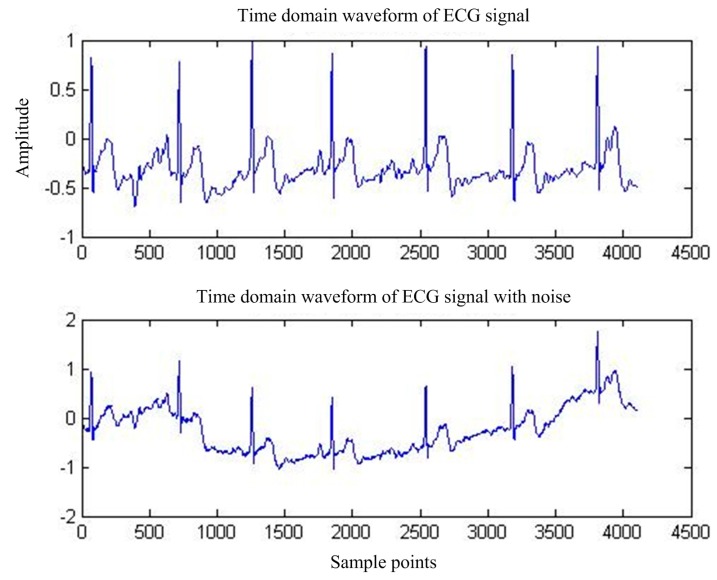
ECG waveforms before and after interference with BW.

**Figure 18:**
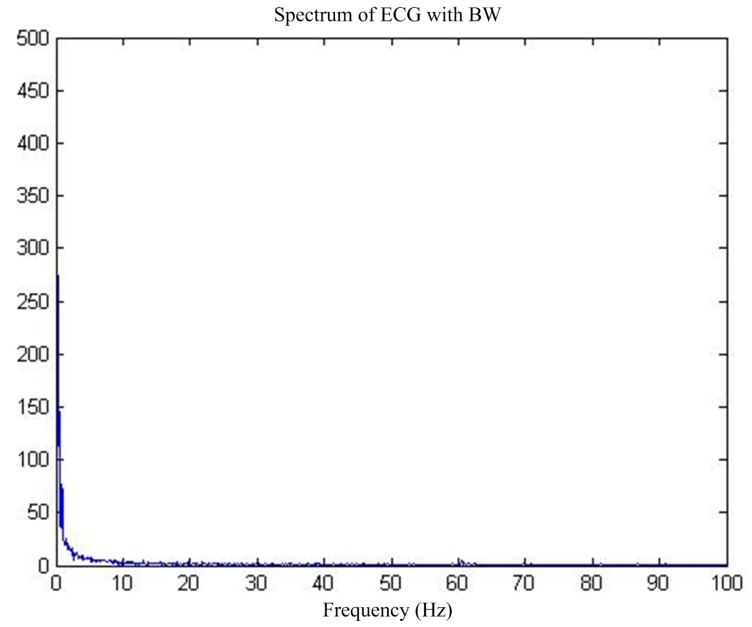
ECG spectrum disturbed by BW.

After verifying the waveforms of the central bank of the public library, the ECG waveforms of a certain classmate are collected for processing using a minicollector. Figure 17 shows the actual measurement of the signal obtained. Figure 18 shows the spectrum.

**Figure 19:**
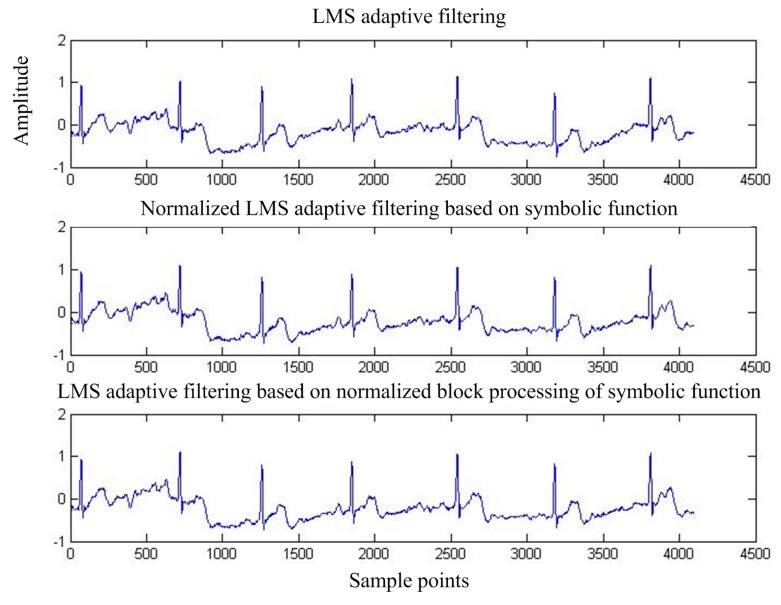
Adaptive interference cancellation after the output signal.

**Figure 20:**
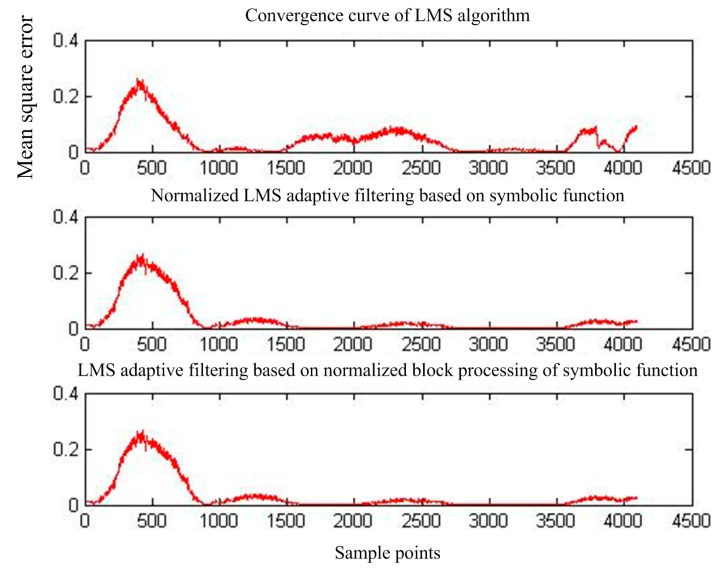
Mean square error convergence of the three adaptive interference cancellation algorithms.

Figure 19 shows the output of adaptive interference cancellation. Figure 20 shows the convergence curve of the statistical mean square error of the three adaptive interference cancellation algorithms.

## 4. Conclusions and Analysis

The basic LMS algorithm, the NLMS algorithm based on symbol function, and the normalized BLMS algorithm based on symbol function are shown in Table 1 for comparison of the computational complexity per L sample points.

As shown in Table 2 and Table 3, these three algorithms can effectively filter out the PLI and obtain the pure ECG waveform at the output. The improved NLMS algorithm and its block-processing algorithm based on symbol function are compared to the LMS algorithm get a higher signal-to-noise ratio (SNR) value.

Table 4 shows the variation of SNR before and after the interference cancellation. It can be seen that the interference cancellation of the three algorithms improves the SNR by 12.5809, 15.8217, and 15.8337 dB, respectively, when the initial SNR is −3.2003 dB.

Table 5 shows the change of SNR before and after the interference cancellation. It can be seen that, under the strong baseline drift interference with the initial SNR of −2.7754 dB, the three adaptive interference cancellation algorithms increase the signal SNR by 13.9228, 16.6120, and 16.6158 dB, respectively.

In summary, the improved symbol-based NLMS algorithm and its block-processing algorithm both improve the SNR both for the central database of the public library and for the analysis of signals from the mini-ECG. Its convergence is reached earlier and eliminating the signal noise interference is better with frequency variation. The improved symbol-based NLMS algorithm has achieved a higher enhancement.

## Figures and Tables

**Table 1:** Comparison of complexity of three kinds of adaptive algorithms.

Algorithm Name	Times of Multiply-Add Operation	Times of Division
Basic LM Salgorithm	*L* + 1	0
NLMS algorithm based on symbol function	1	1
Normalized BLMS algorithm based on symbol function	1	1

**Table 2:** SNR changes of MIT-BIH ECG with PLI before and after denoising.

Algorithm Name	Before Filtering SNR (dB)	After Filtering SNR (dB)	SNRI (dB)
Basic LMS algorithm	−13.5234	19.6638	33.1872
NLMS algorithm based on symbol function	−13.5234	23.3935	36.9168
Normalized BLMS algorithm based on symbol function	−13.5234	23.4859	37.0093

**Table 3:** Changes of SNR before and after denoising of ECG signals from the minicollector with PLI.

Algorithm Name	BeforeFiltering SNR(dB)	AfterFiltering SNB(dB)	SNRI (dB)
Basic LMS algorithm	−12.600	7.4272	20.027
NLMS algorithm based on symbol function	−12.600	10.973	23.572
Normalized BLMS algorithm based on symbol function	−12.600	10.672	23.271

**Table 4:** SNR changes of MIT-BIH ECG signals before and after filtering with BW interference.

Algorithm Name	Before Filtering SNR (dB)	AfterFiltering SNR (dB)	SNRI (dB)
Basic LMS algorithm	−3.2003	9.3806	12.5809
NLMS algorithm based on symbol function	−3.2003	12.6214	15.8217
Normalized BLMS algorithm based on symbol function	−3.2003	12.6334	15.8337

**Table 5:** SNR changes before and after denoising of ECG signals from the minicollector with BW interference.

Algorithm Name	Before Filtering SNR (dB)	After Filtering SNR (dB)	SNRI (dB)
Basic LMS algorithm	−2.7754	11.1474	13.9228
NLMS algorithm based on symbol function	−2.7754	13.8366	16.6120
Normalized BLMS algorithm based on symbol function	−2.7754	13.8404	16.6158
